# Dihydroartemisinin ameliorates the liver steatosis in metabolic associated fatty liver disease mice by attenuating the inflammation and oxidative stress and promoting autophagy

**DOI:** 10.1590/acb385023

**Published:** 2023-10-13

**Authors:** Yiyi Hu, Xuetao Peng, Guoping Du, Yingji Zhai, Xingbo Xiong, Xiaoliang Luo

**Affiliations:** 1 Shunde Hospital of Southern Medical University – Department of Gestroenterology – Foshan – China.; 2 Shunde Hospital of Southern Medical University – Department of VIP Medical Center – Foshan – China.

**Keywords:** Non-Alcoholic Fatty Liver Disease, Fatty Liver, Alcoholic, Inflammation, Oxidative Stress, Autophagy

## Abstract

**Purpose:**

To explore the effect and potential mechanism of dihydroartemisinin (DHA) on metabolism-related fatty liver disease.

**Methods:**

A metabolic associated fatty liver disease (MAFLD) mice model was induced with continuous supplies of high-fat diet. DHA was intraperitoneally injected into mice. The weight of mice was monitored. The concentrations of total cholesterol (TC), triglyceride (TG), low-density lipoprotein (LDL), and high-density lipoprotein (HDL) in serum were detected by an automatic biochemical analyzer. The liver tissues were stained by hematoxylin and eosin and oil red O. The level of inflammation, oxidative stress, and autophagy was assessed by reverse transcription polymerase chain reaction, biochemical examination, Western blot and transmission electron microscope assays.

**Results:**

DHA treatment reduced theMAFLD-enhanced the level of weight gain, the concentrations of TC, TG, LDL and malonaldehyde, while increasedthe MAFLD-decreased the concentrations of HDL and superoxide dismutase. DHA ameliorated the MAFLD-aggravated pathological changes and the number of lipid droplets. Low dose of DHA declined the MAFLD-induced the enhancement of the expression of inflammatory factor. DHA treatment increased the MAFLD-enhanced the level of autophagy related protein, while decreased the MAFLD-reduced the protein level of p62. The increased level of autophagy was confirmed by transmission electron microscope.

**Conclusions:**

DHA can improve liver steatosis in MAFLD mice by inhibiting inflammation and oxidative stress and promoting autophagy.

## Introduction

Metabolic associated fatty liver disease (MAFLD), also known as non-alcoholic fatty liver disease, is a clinicopathological syndrome characterized by diffuse hepatocyte bullous steatosis, which is mainly manifested by liver steatosis, inflammation, progressive liver fibrosis, and ultimately end-stage liver disease^1^,^2^. The occurrence and development of MAFLD are closely related to obesity, insulin resistance, hyperlipidemia, and cardiovascular disease^2^.

A previous study has found that MAFLD is a prerequisite for type-2 diabetes mellitus (T2DM) and metabolic syndrome^3^, and MAFLD occurs in about 50–75% of patients with T2DM^4^. The incidence of MAFLD in normal-weight people is 10–15%, and that in obese people can reach 70%^5^.

MAFLD exists in people of all ages. Love-Osborne et al.^6^ has reported that 53% of obese children had fatty liver, and transaminase in 25% of children is higher than the upper limit of normal. Moreover, the incidence of MAFLD gradually increased as the weight increased. Thus, MAFLD is already the most common liver disease in adults and children worldwide. Therefore, finding effective therapeutic drugs for MAFLD is a problem that needs to be solved in clinic.

There are two main approaches to the pathogenesis of MAFLD, namely the “second strike” theory^7^ and the “multiple strike” theory^8^. The “multiple strikes” theory is a better explanation of the pathogenesis of MAFLD based on the “second strike” theory. Ultimately, it is believed that MAFLD patients are affected by insulin resistance, impaired lipid metabolism, oxidative stress, endoplasmic reticulum stress, mitochondrial dysfunction, nutritional factors, imbalance of gut microbiota, and genetic and epigenetic factors^9^,^10^. As an important organ of lipid metabolism, the liver coordinates fatty acid synthesis, lipid transport, catabolism, and oxidation processes. Abnormalities in any part of the hepatic lipid metabolism process may cause ectopic deposition and abnormal aggregation of lipids, ultimately leading to the occurrence and development of MAFLD^11^.

In addition, oxidative stress is central to the pathogenesis of MAFLD, and there is a close correlation between oxidative stress and abnormal lipid metabolism^12^. Elevated reactive oxygen species (ROS) cause oxidative stress, which triggers lipid peroxidation by targeting the double bond of polyunsaturated FA (PUFA), followed by the formation of 4-hydroxy-2-nonenal (4-HNE) and malondialdehyde (MDA), which reduces the concentration of adenosine triphosphatase (ATP) and nicotinamide dinucleotide. This in turn produces DNA and protein damage, impairs membrane structure and function through lipid peroxidation, and increases the release of pro-inflammatory cytokines causing cellular damage^13^,^14^. Finally, oxidative stress and lipid peroxidation combine to promote the development of MAFLD.

Autophagy, an important cellular process for maintaining cellular homeostasis, enables the cell’s own metabolic needs and the renewal of certain organelles by removing denatured or misfolded proteins, senescent or damaged organelles, which facilitates the maintenance of intracellular homeostasis^15^. Studies have shown that upregulating the level of autophagy in the liver can increase lipid degradation and thus reduce steatosis^16^-^18^. Autophagy is closely related to lipid metabolism, but autophagy is a double-edged sword, and the relationship between autophagy and MAFLD remains controversial.

Dihydroartemisinin (DHA) is a derivative of artemisinin that is widely applied as a first-line antimalarial drug^19^. In addition, plenty of studies have demonstrated that DHA plays the beneficial roles in a variety of disease, such as cancer^20^,^21^, ulcerative colitis^22^, osteoporosis^23^ and pulmonary fibrosis^24^. Furthermore, several reports have confirmed the role of alcoholic liver disease (ALD). Chen et al.^25^ found that DHA notably inhibited ALD via modulation of lipin-1 signaling, as observed that DHA prominently ameliorated hepatocyte lipoapoptosis, hepatocyte and liver injury in chronic alcohol-fed mice. Xu et al.^26^ showed that DHA prevented from alcoholic liver injury through suppressing hepatic steatosis in a farnesoid X receptor-dependent way. Chen et al.^27^ displayed that DHA observably dampened hepatocyte lipoapoptosis by inhibition of PI3K/Akt signaling pathway. However, the effect of DHA on MAFLD and its potential mechanism are still unclear.

Thus, in the present study, we explored the effect of DHA on MAFLD and its potential mechanism. The results showed that DHA improved liver fat deposition in mice with MAFLD via the regulation of autophagy. We hope our results can lay a theoretical basis of the therapy of MAFLD.

## Methods

### Animals

Healthy male C57BL/6 mice (15–25 g, 6 weeks old) were provided by the Experimental Animal Center of Chengdu Dossy Experimental Animals Co. The temperature and relative humidity of the rearing room is 25 ± 2°C and 40–60%, respectively. All experimental procedures were approved and agreed by the Experimental Animal Ethics Committee of West China Hospital, Sichuan University (No. 20221125014).

### Construction of the metabolic associated fatty liver disease mice model

Mice were randomly divided into four groups, including control, MAFLD, MAFLD + DHA-Low, and MAFLD-High. MAFLD mice model was established with high-fat diet for 12 consecutive weeks (60 kcal% Fat, D12492, Dowsontec, China), while mice in control group were fed with standard diet. Ten and 20 mg/kg DHA (Sigma, St. Louis, MO, United States of America) immerged in olive oil (Shanghai Macklin Biochemical Co., Ltd., Shanghai, China) were respectively acted as low- and high-dose of DNA that intraperitoneally injected into mice once daily for five consecutive days per week. Besides, mice in control and MAFLD groups were intraperitoneally injected into the same amount of olive oil. At the end of the experiment, serum and liver tissue samples were isolated and stored for subsequent assays. The animals were sacrificed by cervical dislocation after being anesthetized by an intraperitoneal injection of 2% pentobarbital sodium (35 mg/kg).

### Reverse transcription-quantitative polymerase chain reaction analysis

Total RNA from liver tissue was separated by TRIzol reagent (TaKaRa Biotechnology Co., Ltd., Dalian, China) and reverse transcription (RT) was performed by Bio-Rad ScripTM cDNA Synthesis Kit (Bio-Rad Laboratories, Inc., Hercules, CA, United States of America) based on the manufacturer’s instructions. The resulting RT products were stored at -80°C until analysis. Reverse transcription-quantitative polymerase chain reaction analysis (RT-qPCR) was conducted in a 20-μL mixture incluidng 2 μL of the cDNA templates, 10 μL 2x SYBR Master mix (MedChemexpress, Princeton, NJ, United States of America), 0.4 μL of the 10-μM forward and reverse primers and 7.2 μL ddH_2_O, by the Bio-Rad CFX Manager software (Bio-Rad Laboratories, Inc.). The RT-qPCR conditions were as follows: 5 min at 94°C, followed by 40 cycles between 94°C for 15 s and 58°C for 30 s, and 72°C for 30 s. The relative expressions of tumor-necrosis factor (TNF)-α (Forward primer: 5’- GCGGTGCCTATGTCTCAGCCTCTTCT -3’, Reverse primer: 5’- GGTGGTTTGTGAGTGTGAGGGTCTGG -3’), interleukin (IL)-6 (Forward primer: 5’- GTATGAACAGCGATGATGCACT -3’, Reverse primer: 5’- GTATGAACAGCGATGATGCACT -3’), and IL-8 (Forward primer: 5’- GGACCACAACCACTGCGCCAACACAGAA -3’, Reverse primer: 5’- GGCAACCCTACAACAGACCCACACAA -3’) were analyzed using the 2^-ΔΔCT^ method and normalized to the housekeeping gene β*-actin*. The primer sequences were synthesized in Sangon Biotech Co. (Shanghai, China).

### Biochemical examination

The level of high-density lipoprotein (HDL), total cholesterol (TC), triglyceride (TG) and low-density lipoprotein (LDL) in serum were examined with an automatic biochemical analyzer (model 7150; Hitachi, Tokyo, Japan) according to the manufacturer’s instructions.

The concentration of superoxide dismutase (SOD) and MDA were measured using commercial total superoxide dismutase (T-SOD) test kit (A001-1-1, Nanjing Jiancheng Bioengineering Institute, Nanjing, China) and MDA test kit (A003-1-1, Nanjing Jiancheng Bioengineering Institute) according to the operating manual. The absorbance of wells was determined at 560 nm (SOD) and 532 nm (MDA) using a microplate reader (Thermo Fisher Scientific), respectively.

### Western blot analysis

Protein from liver tissue was lysed using RIPA lysis buffer (Boster, Wuhan, China). Subsequently, protein samples (20 μg) were isolated by 10% SDS-PAGE and then electrically transferred onto polyvinylidene fluoride (PVDF) membranes (EMD Millipore, Billerica, MA, United States of America). The membranes were blocked in 5% skimmed milk powder for 1 hour at room temperature, and then hatched with primary antibodies at 4°C overnight, followed by incubation with the corresponding secondary antibody (Boster) for 1 hour at room temperature. The bands were visualized using an ECL chemiluminescence kit (EMD Millipore) based on the manufacturer’s instructions. The gray value was analyzed using Image-ProPlus software (Media Cybernetics, Inc., Rockville, MD, United States of America). Protein levels were determined relative to β-actin. The primary antibodies used were as follows: p62 (5114), Beclin-1 (3738), LC3I /LC3II (4108), PINK1 (6946), Parkin (2132), and β-actin (4970; Cell Signaling Technology, Inc., Danvers, MA, USA) at 1:1,000 dilution.

### Histological analysis

Liver tissues were separated, fixed in 4% formaldehyde, dehydrated, embedded, and cut into sections. Then, sections were stained with hematoxylin and eosin (H&E). The stained sections were captured by a light microscope (Olympus, Tokyo, Japan), and images were analyzed by Image-Pro Plus 6.0 software (Media Cybernetics, United States of America).

Liver tissues were fixed in 4% paraformaldehyde for 10 min, followed by staining with oil red O for 30 min. Then, the cells were dyed with hematoxylin and differentiated at 1% hydrochloric acid alcohol (rapid). Images were captured under a light microscope (Olympus).

### Transmission electron microscopy

Liver tissues were fixed in 3% glutaraldehyde and 1% osmium tetroxide and then cut on an ultramicrotome. Subsequently, sections were stained with 1% uranyl acetate and 0.5% lead citrate successively. The images were analyzed using JEM-1400PLUS transmission electron microscope.

### Statistical analysis

Statistical analysis was carried out using Statistical Package for the Social Sciences 20.0 software (IBM Corp., Armonk, NY, United States of America). All data are expressed as mean ± standard deviation. The Student’s t-test was used to compare the data with only two groups, while the one-way analysis of variance was applied to determine the differences among multiple groups. The differences were thought as statistically significant when p < 0.05.

## Results

### Dihydroartemisinin treatment improved the liver steatosis in metabolic associated fatty liver disease mice

**Figure 1:**
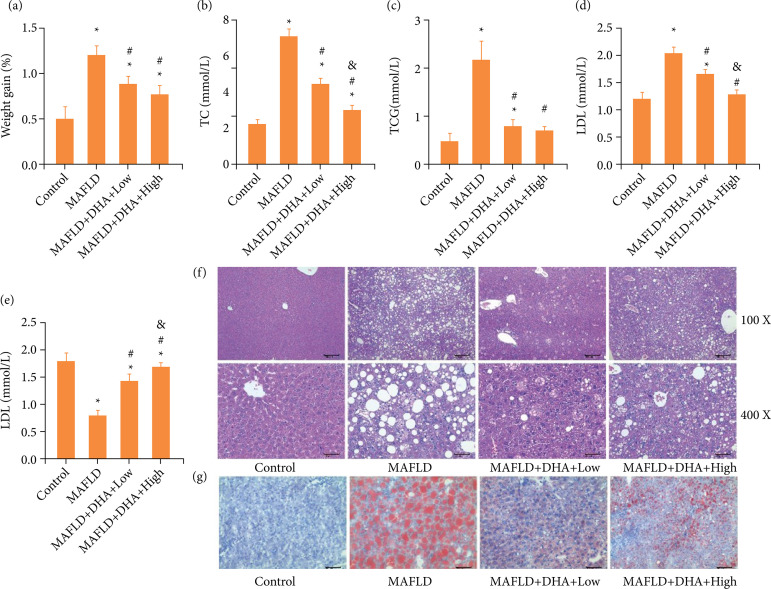
DHA treatment relieved the liver steatosis in MAFLD mice. **(a)** The weight of mice in each group was monitored continuously, and then the weight gain was calculated. The concentrations of **(b)** TC, **(c)** TG, **(d)** LDL, **(e)** and HDL in serum were analyzed by an automatic biochemical analyzer. **(f)** The live tissue samples were stained by hematoxylin and eosin. **(g)** The live tissue samples were stained by oil red O.

DHA: dihydroartemisinin; MAFLD: metabolic associated fatty liver disease; TC: total cholesterol; TG: triglyceride; LDL: low-density lipoprotein; HDL: high-density lipoprotein; *p < 0.05 compared to control group; #p < 0.05 compared to MAFLD group; &p < 0.05 compared to MAFLD-Low group. Source: Elaborated by the authors.

Mice were induced with high-fat diet continuously to form the MAFLD model, and then intraperitoneally treated with low- or high-dose of DHA. The weight gain (Fig. 1a), concentrations of TC (Fig. 1b), TG (Fig. 1c) and LDL (Fig. 1d) of MAFLD mice were significantly increased compared to these in control mice, while the concentrations of HDL (Fig. 1e) of MAFLD mice was prominently decreased relative to that in control mice. Besides, H&E staining results (Fig. 1f) showed diffuse steatosis in the liver tissue, the liver lobules almost replaced by large transparent round lipid droplets, the increase of storage cells, balloon-like changes of liver cells in some areas, and a small amount of inflammatory cell infiltration in the portal area. Also, the number of lipid droplets stained red by oil red O (Fig. 1g) was notably enhanced compared with that in control group.

Thus, these results indicated that the MAFLD model was built in mice successfully. However, both low- and high-dose of DHA observably reduced the MAFLD-induced the elevation of weight gain (Fig. 1a), concentrations of TC (Fig. 1b), TG (Fig. 1c) and LDL (Fig. 1d), while enhanced the MAFLD-decreased the concentrations of HDL (Fig. 1e). Moreover, both low- and high-dose of DHA also ameliorated the MAFLD-aggravated pathological changes (Fig. 1f) and the number of lipid droplets (Fig. 1g). In addition, statistical differences were observed in the concentrations of TC (Fig. 1b), LDL (Fig. 1d) and HDL (Fig. 1e) between MAFLD-Low and MAFLD-High groups. Taken together, these results suggested that a successful MAFLD model were constructed in mice, and DHA treatment ameliorated the liver steatosis in MAFLD mice.

### Dihydroartemisinin treatment alleviated the inflammation and oxidative stress in metabolic associated fatty liver disease mice

**Figure 2:**
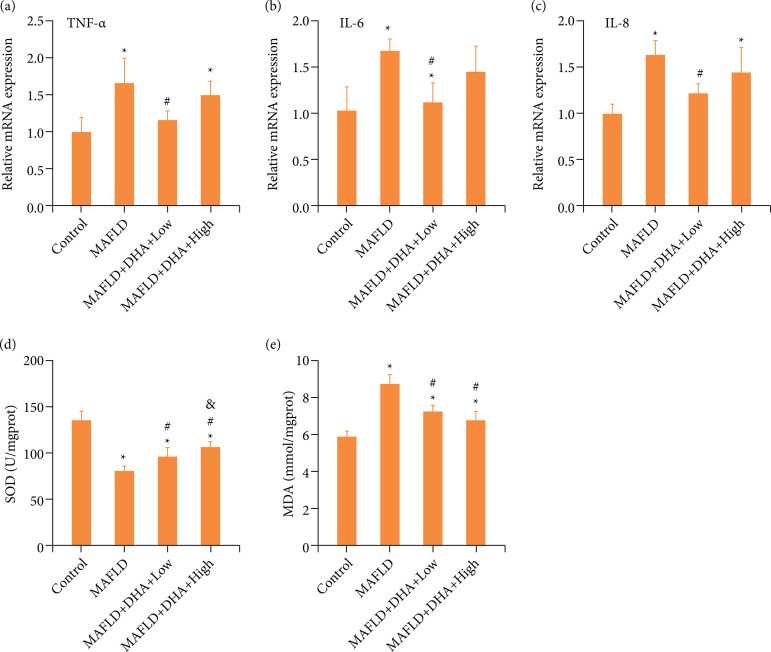
DHA treatment lightened the inflammation and oxidative stress in MAFLD mice. The relative expressions of **(a)** TNF-α, **(b)** IL-6, and **(c)** IL-8 were detected by quantitative reverse transcription polymerase chain reaction. The data were expressed after being normalized to β*-actin*. (d and e) The concentrations of SOD and MDA were determined by commercial kits.

DHA: dihydroartemisinin; MAFLD: metabolic associated fatty liver disease; TNF: tumor-necrosis factor; IL: interleukin; SOD: superoxide dismutase; MDA: malondialdehyde; *p < 0.05 compared to control group; #p < 0.05 compared to MAFLD group; &p < 0.05 compared to MAFLD-Low group. Source: Elaborated by the authors.

The potential mechanisms involved in the improvement of DHA treatment in the liver steatosis of MAFLD mice were explored. Quantitative reverse transcription polymerase chain reaction (qRT-PCR) results revealed that all the relative expressions of TNF-α (Fig. 2a), IL-6 (Fig. 2b) and IL-8 (Fig. 2c) were markedly enhanced compared with these in control group, which indicated that MAFLD leaded to inflammation in mice. Besides, the concentration of SOD (Fig. 2d) was signally declined with memorably increased the concentration of MDA (Fig. 2e) in MAFLD mice, which also suggested that MAFLD resulted in oxidative stress in mice. However, only low dose of DHA significantly reversed the MAFLD-induced the enhancement of relative expressions of TNF-α (Fig. 2a), IL-6 (Fig. 2b) and IL-8 (Fig. 2c), whereas both low and high dose of DHA prominently inverted the MAFLD-elicited the alteration of SOD (Fig. 2d) and MDA (Fig. 2e). Statistical difference was just observed in concentration of SOD between MAFLD-Low and MAFLD-High groups (Fig. 2d). Therefore, these results illustrated that DHA treatment mitigated the inflammation and oxidative stress in MAFLD mice.

### Dihydroartemisinin treatment ameliorated the liver steatosis in metabolic associated fatty liver disease mice via autophagy

**Figure 3:**
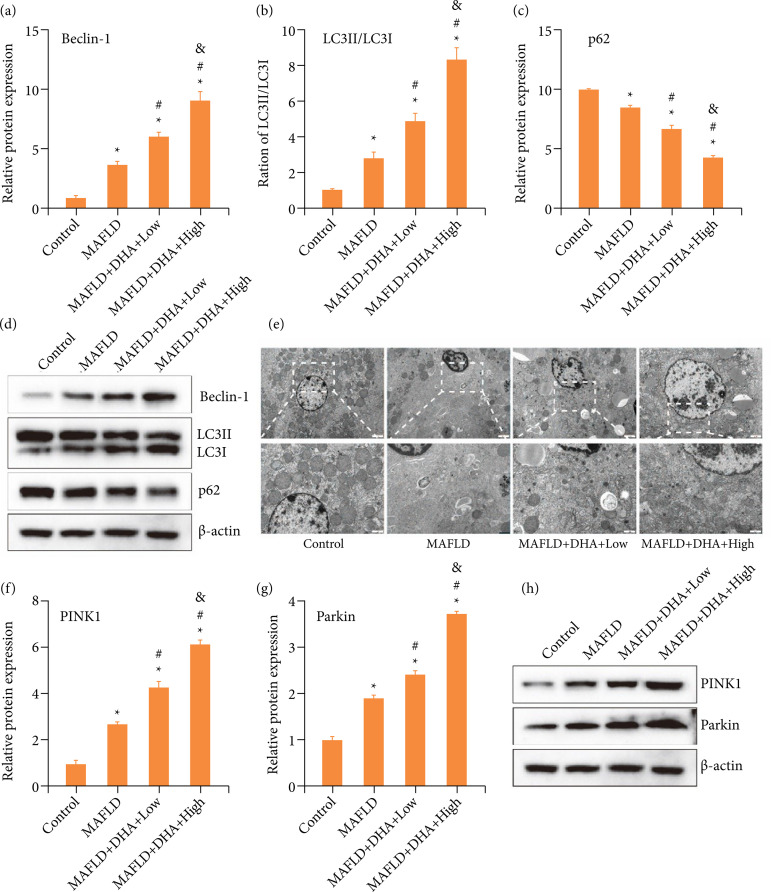
**DHA treatment improved the liver steatosis in MAFLD mice via autophagy. (a–c)** The relative protein level of Beclin-1, LC3 and p62 was examined by Western blot. The data were expressed after being normalized to β-actin. **(d)** The relative intensity of the Beclin-1, LC3 and p62 proteins was shown as a bar graph. **(e)** The autophagosome in liver tissue samples was assessed by transmission electron microscope. (f and g) The relative protein level of PINK1 and Parkin was detected by Western blot. The data were expressed after being normalized to β-actin. **(h)** The relative intensity of the PINK1 and Parkin proteins was shown as a bar graph.

DHA: dihydroartemisinin; MAFLD: metabolic associated fatty liver disease; *p < 0.05 compared to control group; #p < 0.05 compared to MAFLD group; &p < 0.05 compared to MAFLD-Low group. Source: Elaborated by the authors.

Moreover, the role of autophagy in the alleviation of DHA treatment in the liver steatosis of MAFLD mice was also investigated. Western blot results showed that both low and high dose of DHA further notably increased the MAFLD-enhanced the relative protein level of Beclin-1 (Figs. 3a and 3d) and LC3 (Figs. 3b and 3d), while further observably decreased the MAFLD-reduced the relative protein level of p62 (Figs. 3c and 3d). Furthermore, both low and high dose of DHA distinctly improved the MAFLD-induced cell necrosis, chromatin aggregation, fragmented nuclei, blurred structure, disordered cytoplasmic content, pyknotic mitochondria, and the increase of autophagosomes (Fig. 3e). In addition, the relative protein level of PINK1 (Figs. 3f and d) and Parkin (Figs. 3g and 3d) was notably elevated in MAFLD mice, which was further augmented with both low and high dose of DHA treatment. Statistical differences were all observed in the relative protein level of Beclin-1, LC3, p62, PINK1 and Parkin between MAFLD-Low and MAFLD-High groups. Thus, these results suggested that DHA treatment alleviated the liver steatosis in MAFLD mice via autophagy.

## Discussion

MAFLD is a common chronic liver condition which is closely related in cardiovascular disease, T2DM and metabolic syndrome 2.DHA is a famous antimalarial drug, whose beneficial roles in ALD have been demonstrated in the previous studies^25^-^27^.

In the present study, mice were fed with high-fat diet continuously to generate the MAFLD model, which was confirmed by the increase of the concentrations of TC, TG and LDL with the decrease of HDL, H&E staining, as well as oil red O staining. DHA treatment could notably improve the MAFLD in mice through the reduction of indicators of inflammation, oxidative stress, and autophagy. Thus, we concluded that DHA treatment ameliorated the liver steatosis in MAFLD mice via inhibiting the inflammation, oxidative stress and promoting autophagy.

The MAFLD animal model induced by a high-fat diet can trigger further obesity, metabolic syndrome, and insulin resistance^28^. The high-fat diet-induced MAFLD model has been widely used in plenty of reports^29^-^31^. Similar to these previous studies, a MAFLD mice model was induced with continuous supplies of high-fat diet for 12 weeks. We found that the weight gain, and concentrations of TC, TG and LDL were significantly increased with the decreased the concentrations of HDL in MAFLD mice. As the common indicators of blood lipid, the dysregulation of TC, TG, LDL and HDL is regarded as the risk factor of blood lipid^32^.

Thus, combined with the results from pathologic changes and oil red O staining, we concluded that a MAFLD mice model was successfully built in the present study. However, DHA treatment observably reduced the MAFLD-enhanced the weight gain, and concentrations of TC, TG and LDL and increased the MAFLD-decreased the concentrations of HDL. Moreover, DHA treatment also ameliorated the MAFLD-aggravated pathological changes and the number of lipid droplets. Therefore, these results indicated that DHA treatment ameliorated the liver steatosis in MAFLD mice.

Mechanically, DHA treatment alleviated the inflammation and oxidative stress in MAFLD mice, as shown by DHA treatment significantly reversed the MAFLD-induced the enhancement of relative expressions of TNF-α, IL-6 and IL-8, and the concentration of MDA with prominently inverted the MAFLD-declined the concentration of SOD. Growing evidence has revealed that MAFLD is tightly involved in the inflammation and oxidative stress^33^-^35^.

TNF-α, IL-6 and IL-8 are common pro-inflammatory cytokines, whose level effectively reflex the degree of inflammatory response^36^. MDA is a degradation production of lipid peroxides that indicates the peroxidation level of body fat^37^. As an swept away, SOD is an antioxidant enzyme that can clear oxygen free radicals to modulate the balance of oxidation and antioxidant, and also converse superoxide anion free radicals into hydrogen peroxide to further transform into water by GSH-Px^37^.

Moreover, the anti-inflammation and anti-oxidative stress roles of DHA have been exhibited in a variety of disease models. For instance, Liu et al. showed that DHA alleviated lipopolysaccharide (LPS)-triggered acute kidney injury via suppressing inflammation and oxidative stress^38^. Similar anti-inflammation and anti-oxidative stress effect of DHA has been also displayed in the LPS-elicited acute lung injury mice model^39^. Consistent with these studies, our results also elucidated that DHA treatment mitigated the inflammation and oxidative stress in MAFLD mice.

In addition, autophagy has been also demonstrated to be one of major pathogenesis of MAFLD^40^. Zhang et al. showed that galangin ameliorated MAFLD via enhancing autophagy^41^. Stacchiotti et al. reported that autophagy was associated with the effect of melatonin on MAFLD^42^. Yang et al. exhibited that naringenin dampened MAFLD via the modulation of autophagy^43^. Similarly, the regulated roles of DHA were also involved in autophagy, which has been reported in various models, such as balloon injury-induced neointimal formation^44^, cancers^45^, hypertrophic scars formation^46^, and catabolism in chondrocytes^47^.

In the present study, DHA treatment notably increased the MAFLD-enhanced the relative protein level of Beclin-1, LC3, PINK1 and Parkin, while further observably decreased the MAFLD-reduced the relative protein level of p62. Also, the increased level of autophagy was further confirmed by transmission electron microscope observation. PINK1 is a mitochondrial serine/threonine protein kinase that plays an important role in regulating mitochondrial dynamics, trafficking, and quality control^48^. Parkin is the E3 ubiquitin ligase that also modulates the mitochondrial quality control. Furthermore, Parkin can accelerate the PINK1-directed autophagic clearance of depolarized mitochondria^49^.

Taken together, these results suggested that DHA treatment alleviated the liver steatosis in MAFLD mice via promoting autophagy.

## Conclusion

In conclusion, our data indicated that DHA treatment ameliorated the liver steatosis in MAFLD mice via inhibiting the inflammation, oxidative stress and promoting autophagy. We hope our findings can lay a foundation of the development of therapeutic drugs for MAFLD.

## Data Availability

The datasets used or analyzed during the current study are available from the corresponding author.
